# Supplementary material for: The genome sequence of the silvery leafcutter bee, Megachile leachella Curtis, 1828
Source: Wellcome Open Res. 2024 Jul 29;9:415. [Version 1] doi: 10.12688/wellcomeopenres.22619.1 (PMC11574336; doi:10.12688/wellcomeopenres.22619.1)
Supplement: Peer review history

# Reviewer report: Reviewer response for version 1

**DOI:** 10.21956/wellcomeopenres.24919.r103806

**Published:** 2024-11-18

**Authors:** Alexis L Sperling

**Affiliations:** 1 University of Cambridge, Cambridge, England, UK

This report describes that genome assembly and sequence of the leafcutter bee, *Megachile leachella*, as part of the Darwin Tree of Life project. It is a good, technically sound report with all the necessary data to be useful for anyone doing comparative genomics or those looking into the fascinating biology of these bees.

 I believe that there could be a little more information about the bee in the introduction. For example, is there more known about their conservation or if they are threatened the way that other bee species are?

 Figure 1 shows a pictures of the bee, which is standard for these reports but there is also a one of the specimen in the wild which is nice. From table 1 and the snail plot in figure 2, the assembly is excellent, with a a very high Consensus quality (QV). It might be nice to know how this assembly matches up with other bee assemblies. They could discuss the sex chromosomes and why they don't identify any. I am not a fan of BlobToolKit analysis, Figures 3/4, but this one show what is expected, that mostly scaffolds are Arthropoda. The contact map in figure 5 shows that the assembly is likely correct. The chromosome table (table 2) shows the details for each chromosome, they could add a little info about why the GC content goes down with chromosome size.

 Taken together this is a good report which would only benefit from a little more background and interpretation of the data.

Are sufficient details of methods and materials provided to allow replication by others?

Yes

Is the rationale for creating the dataset(s) clearly described?

Yes

Are the datasets clearly presented in a useable and accessible format?

Yes

Are the protocols appropriate and is the work technically sound?

Yes

Reviewer Expertise:

Parthenogenesis.

I confirm that I have read this submission and believe that I have an appropriate level of expertise to confirm that it is of an acceptable scientific standard.

# Reviewer report: Reviewer response for version 1

**DOI:** 10.21956/wellcomeopenres.24919.r103808

**Published:** 2024-11-02

**Authors:** Jiangli Tan

**Affiliations:** 1 Northwest University, Xi An, China

It is a paper on a genome assembly from the silvery leafcutter bee *Megachile leachella* (Insecta; Hymenoptera; Megachilidae) with positive results. 

 The language is good enough and the sequencing data and assembly is excellent. However, there are still some parts can improve.

1. In introduction or discussion, should mention the research background on other insect species especiely on the species of the same genus or family. Compare with other similar research is neccessary on understanding your research innovation.
2. Discussion part should not ignore. At least discuss your sequence data is somewhere special or useful.
3. Suggestion: GPS better as (N..., W...)
4. Species Taxonomy part is redundant。 Only （Insecta, Hymenoptera, Apocrita, Aculeata, Apoidea, Megachilidae, Megachilinae, *Megachile leachella* Curtis, 1828 (NCBI: txid1542539). is enough.
5. Figure 4 is too big compare its poor information showing. 

Are sufficient details of methods and materials provided to allow replication by others?

Yes

Is the rationale for creating the dataset(s) clearly described?

Yes

Are the datasets clearly presented in a useable and accessible format?

Yes

Are the protocols appropriate and is the work technically sound?

Yes

Reviewer Expertise:

Taxonomy on entomology

I confirm that I have read this submission and believe that I have an appropriate level of expertise to confirm that it is of an acceptable scientific standard, however I have significant reservations, as outlined above.

# Reviewer report: Reviewer response for version 1

**DOI:** 10.21956/wellcomeopenres.24919.r103804

**Published:** 2024-10-18

**Authors:** Christopher Wyatt

**Affiliations:** 1 University College London, London, England, UK

A high-quality genome sequence of *M. leachella* has been presented in a clear and straight forward article. The genome was sequenced from a female bee, yielding 62x coverage using HiFi long reads and Hi-C data for scaffolding to obtain a chromosome level assembly, which appears at the same quality as previously published bee genomes. 

 I could not see any faults in this article, it is exciting to have this genome available to the public, but just had two questions:

1.  Chromosome-scale scaffolds were assigned, covering 56.52% of the genome. Is there a reason why not as much of the genome was captured within HiC chromosomes?
2.  Also, the length of the mitochondrial genome seems different, most other Megachile species have a ~16-17 kilobase length ( Zhang Y et al. (2017 ^1^)), whereas yours is reported much longer than the average. Is there a reason for this?

Are sufficient details of methods and materials provided to allow replication by others?

Yes

Is the rationale for creating the dataset(s) clearly described?

Yes

Are the datasets clearly presented in a useable and accessible format?

Yes

Are the protocols appropriate and is the work technically sound?

Yes

Reviewer Expertise:

omics, evolution

I confirm that I have read this submission and believe that I have an appropriate level of expertise to confirm that it is of an acceptable scientific standard.

## References

[1] : Sequencing and characterization of the Megachile sculpturalis (Hymenoptera: Megachilidae) mitochondrial genome. *Mitochondrial DNA A DNA Mapp Seq Anal* .2017;28(3) : 10.3109/19401736.2015.1122774 344-346 10.3109/19401736.2015.1122774 26714001
